# Case report: surgical total cardiac denervation for refractory vasospastic angina: first report with pre- and post-operative autonomic assessment

**DOI:** 10.1093/ehjcr/ytag691

**Published:** 2026-10-14

**Authors:** Mitchell Hobers, Erik Lipsic, Riemer H J A Slart, Mike J L de Jongste, Theo J Klinkenberg

**Affiliations:** Department of Cardiology, University Medical Center Groningen, Hanzeplein 1, 9713 GZ Groningen, The Netherlands; Department of Cardiology, University Medical Center Groningen, Hanzeplein 1, 9713 GZ Groningen, The Netherlands; Department of Cardiology, University Medical Center Groningen, Hanzeplein 1, 9713 GZ Groningen, The Netherlands; Department of Cardiology, University Medical Center Groningen, Hanzeplein 1, 9713 GZ Groningen, The Netherlands; Department of Cardiology, University Medical Center Groningen, Hanzeplein 1, 9713 GZ Groningen, The Netherlands

**Keywords:** Vasospastic angina, Autonomic nervous system, ^123^I-MIBG, HRV, Surgical total cardiac denervation, Case report

## Abstract

**Background:**

Refractory vasospastic angina (VSA) can persist despite maximal medical therapy and partial autonomic denervation. Surgical total cardiac denervation (STCD) is a novel surgical approach resulting in disappearance of the anginal pain perception.

**Case summary:**

We report the first clinically successful STCD supported by objective pre- and post-operative autonomic assessment using heart rate variability (HRV) and iodine-123 metaiodobenzylguanidine (^123^I-MIBG) scintigraphy. A 53-year-old woman with severe treatment-refractory VSA remained highly symptomatic. Pre-operative HRV and ^123^I-MIBG both demonstrated preserved autonomic innervation. Following STCD, anginal symptoms resolved and ST-T segment changes became painless. Concomitantly, HRV markedly decreased and ^123^I-MIBG showed significantly reduced tracer uptake, confirming complete sympathetic denervation. After a 9-month follow-up, the patient remains free of symptoms.

**Conclusion:**

We present the first proof-of-concept case of STCD as a novel approach for refractory VSA. Further experience is required to evaluate broader efficacy, as well as long-term durability and safety.

Learning points

- Surgical total cardiac denervation (STCD) is a novel surgical strategy aimed at eliminating autonomic nervous effect.
- Objective autonomic assessment can document the presence or absence of cardiac sympathetic innervation before and after denervation procedures.
- STCD may be considered as a last-resort option in patients with refractory vasospastic angina who fail maximal medical therapy and partial denervation interventions.

## Introduction

Vasospastic angina (VSA) is characterized by transient epicardial coronary vasoconstriction. The vasoconstriction may induce myocardial ischaemia, angina, and potentially malignant ventricular arrhythmias.^1,2^ Most patients benefit from vasodilators; however, some continue to experience severe angina with a serious impact on daily quality of life. Autonomic dysregulation contributes to vasospasm pathophysiology through both efferent sympathetic pathways—mediating vasoconstriction via direct and paracrine mechanisms—and afferent pathways convey ischaemic chest pain signals.^3,4^

Partial denervation procedures such as renal sympathetic denervation or thoracoscopic sympathectomy can offer temporary benefit,^5^ but neural regrowth or incomplete interruption frequently results in symptom recurrence.

Surgical total cardiac denervation (STCD) is a novel surgical approach intended to eliminate major extrinsic autonomic pathways entering the heart. In contrast to auto-transplantation-based denervation,^6^ STCD does not have the disadvantage of a cardiac explantation, while it also exhibits complete extensive anatomical and electrophysiological denervation.

To the best of our knowledge, this is the first report of STCD as a therapeutic strategy for refractory VSA, supported by objective autonomic assessment both before and after surgery.

## Summary figure

**Figure:**
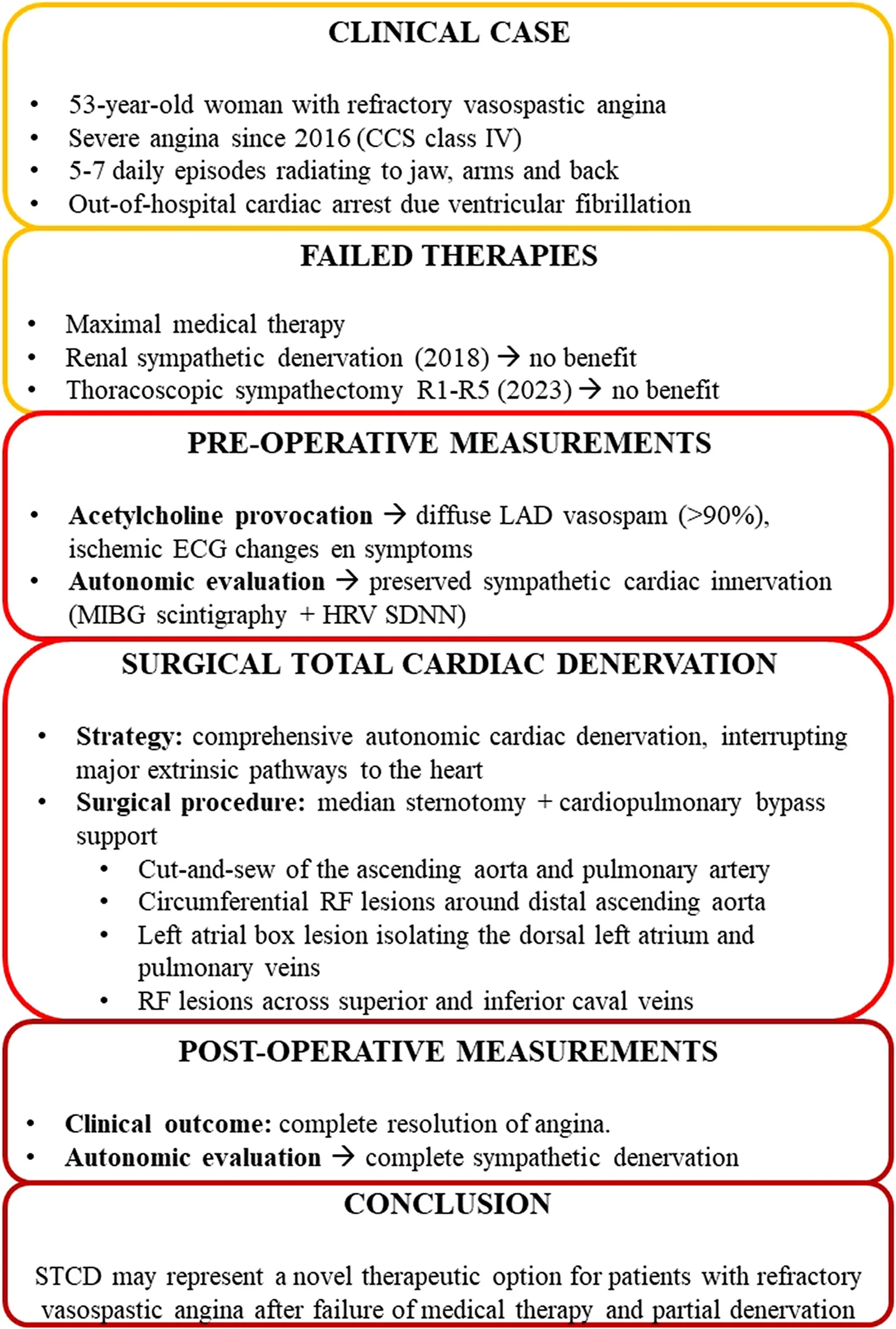


## Case presentation

A 53-year-old woman was referred from a regional hospital to our tertiary University centre for evaluation of refractory VSA. She had experienced severe angina since 2016 [Canadian Cardiovascular Society (CCS) Class IV], with 5–7 daily episodes radiating to jaw, arms, and back. Initially, symptoms were predominantly triggered by physical exertion; however, over time, episodes also occurred at rest without identifiable precipitating factors. At the onset of her disease in 2016, she experienced an out-of-hospital cardiac arrest due to ventricular fibrillation (VF) triggered by coronary vasospasm. Prior to this event, she had not received specific antianginal or antiarrhythmic therapy, and no previous ventricular arrhythmias were documented. A single-chamber implantable cardioverter-defibrillator was implanted for secondary prevention.

Despite maximal tolerated medical therapy—including nifedipine, isosorbide mononitrate, a nitroglycerin patch, ivabradine, verapamil, ramipril, acetylsalicylic acid, pravastatin, ezetimibe, and even paracetamol and oxycodone—she continued to experience CCS Class IV angina. The patient was fully adherent to the prescribed medical therapy, which was well tolerated without symptomatic hypotension (blood pressure 130/70 mmHg), and there was no evidence that hypotension contributed to her persistent anginal symptoms. In 2018 she underwent renal sympathetic denervation as an experimental attempt to reduce sympathetic tone. The procedure was completed successfully despite three intraprocedural cardiac arrests requiring extracorporeal life support. However, no sustained clinical benefit was observed. In 2023, she underwent a left thoracoscopic sympathectomy (R1–R5) performed by the cardiothoracic surgery team, with initial symptomatic improvement followed by recurrence of frequent anginal episodes within months.

At our outpatient cardiology clinic, the symptoms were reviewed and a complete diagnostic work-up was performed, including heart rate variability (HRV) assessment by means of 24-h ambulatory ECG recording, using the standard deviation of normal-to-normal intervals (SDNN) as a marker of cardiac autonomic modulation, and iodine-123 metaiodobenzylguanidine (^123^I-MIBG) scintigraphy to assess cardiac sympathetic innervation (*Figure 1*), and coronary angiography with intracoronary flow measurements.

**Figure 1:**
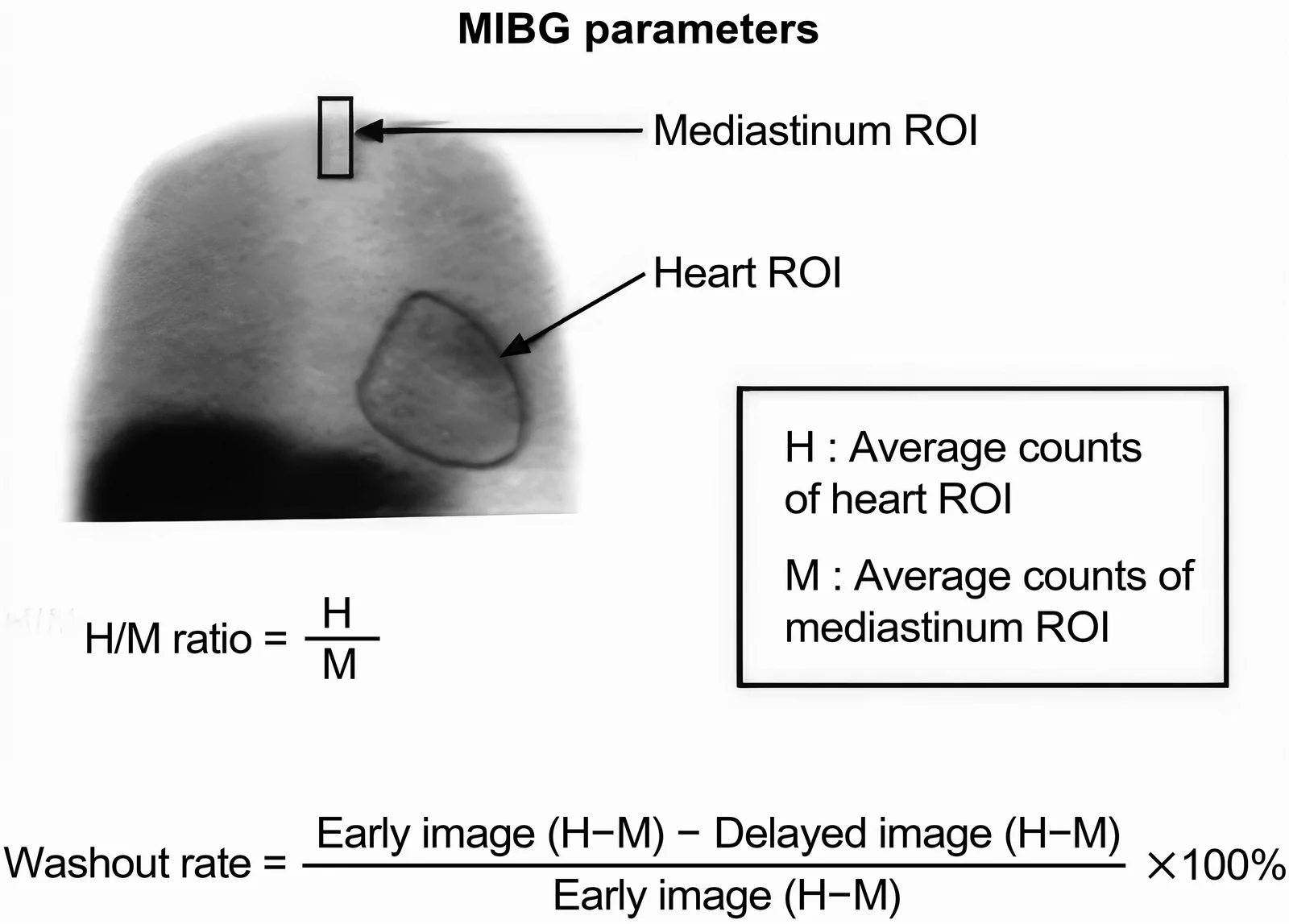
MIBG parameters. MIBG, metaiodobenzylguanidine; ROI, regions of interest; H, heart; M, mediastinum. The late heart-to-mediastinum (H/M) ratio reflects cardiac sympathetic innervation. Higher values indicate preserved sympathetic nerve activity, whereas markedly reduced values indicate impaired or absent sympathetic innervation. Reference values depend on imaging methodology; in this case, the pre-operative H/M ratio of 2.55 was consistent with preserved sympathetic innervation, while the post-operative value of 0.82 indicated severe sympathetic denervation.

Pre-operative HRV (SDNN 110 ms) and ^123^I MIBG (late Heart/Mediastinum ratio 2.55) demonstrated preserved cardiac autonomic innervation. Coronary angiography with intracoronary acetylcholine provocation (100 mcg) induced diffuse epicardial LAD vasospasm (>90%) with ST-T segment changes and reproduction of symptoms, followed by full resolution after intracoronary nitroglycerin administration (*Figure 2*). Coronary physiological assessment was subsequently performed using a PressureWire™ X Guidewire (Abbott), demonstrating a coronary flow reserve of 8.5 and an index of microvascular resistance of 11, both within normal limits, consistent with the absence of microvascular dysfunction.

**Figure 2:**
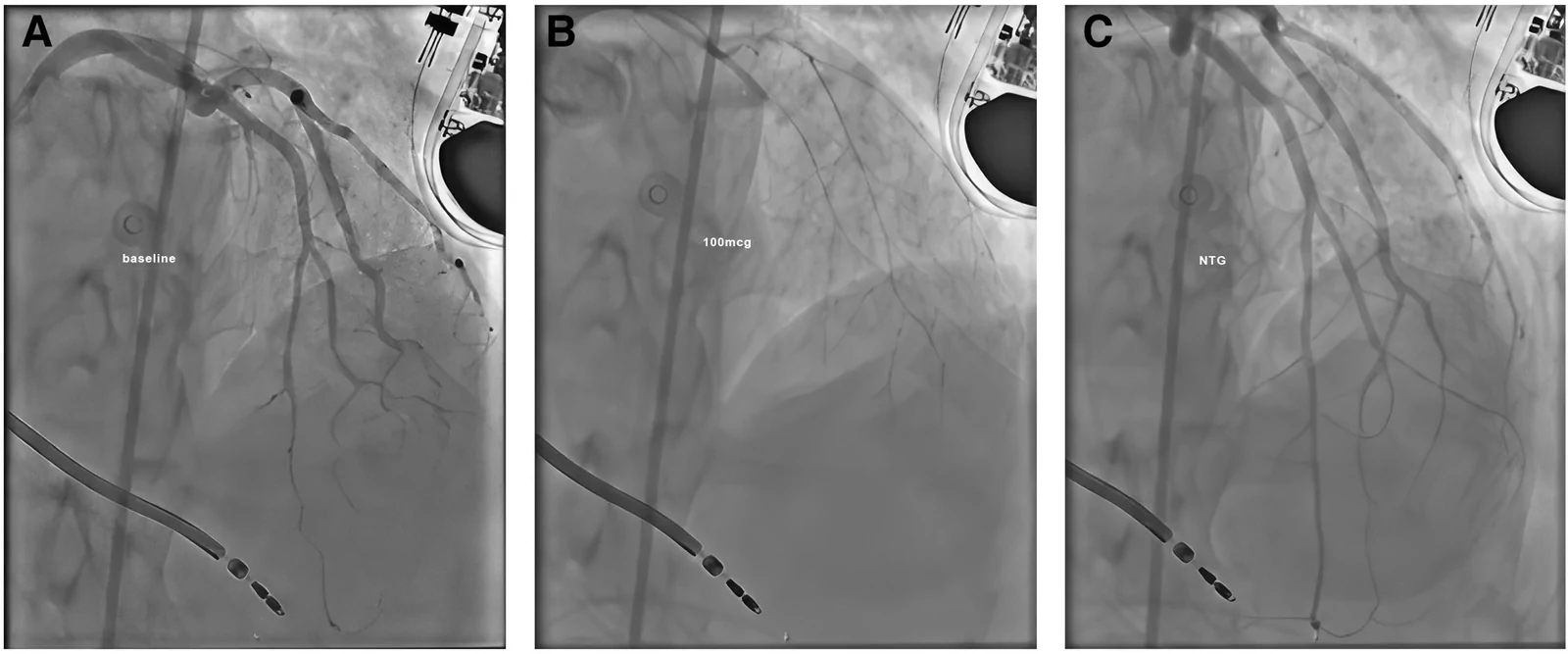
Coronary angiography with acetylcholine provocation testing. *A*: Baseline coronary angiography demonstrating a normal left anterior descending artery (LAD). *B*: Following intracoronary administration of 100 μg acetylcholine, diffuse severe (>90%) epicardial LAD vasospasm developed and reproduction of the patient's typical anginal symptoms. *C*: Complete angiographic resolution after intracoronary nitroglycerin administration.

After reviewing these findings, which were consistent with the standardized diagnostic criteria for VSA, she was discussed in the local heart team (cardiac surgeon and interventional cardiologist) and accepted for STCD as a last-resort treatment option.

### Surgical procedure

STCD was performed via median sternotomy with cardiopulmonary bypass support. The procedure, which lasted 3 h and 54 min, combined anatomical transection with extensive radiofrequency (RF) lesion sets to eliminate major extrinsic autonomic pathways.

The denervation process began with cut-and-sew transection of the ascending aorta and the central pulmonary artery, interrupting the dense perivascular sympathetic plexus surrounding these vessels. The distal ascending aorta was then circumferentially treated with RF energy using the Isolator® Synergy™ Clamp (AtriCure Inc.), creating a continuous transmural lesion of at least 1 cm in width to minimize the potential for sympathetic nerve regrowth (*Figure 3*).

**Figure 3:**
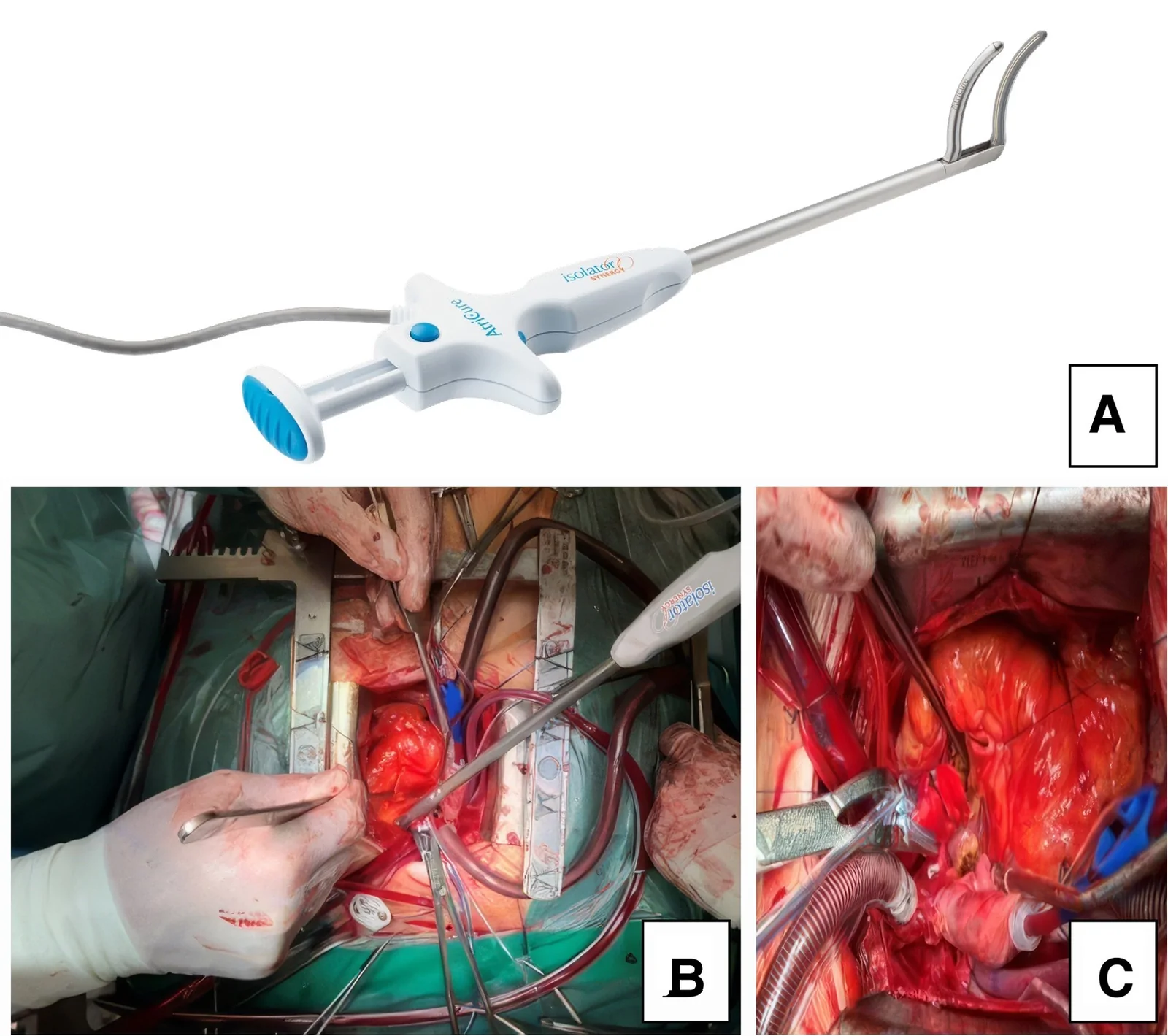
Surgical total cardiac denervation procedure. *(A*) Isolator® Synergy™ Clamp. *(B*) The distal ascending aorta is circumferentially treated with RF energy using the Isolator® Synergy™ Clamp (AtriCure Inc.), creating a continuous transmural lesion of at least 1 cm in width to minimize the potential for sympathetic nerve regrowth. *(C*) A cut-and-sew transection of the ascending aorta and the central pulmonary artery with the distal lesion set below the aortic cross clamp.

A left atrial box lesion isolating the dorsal left atrium and pulmonary veins was created using the EnCompass® Synergy™ Clamp (AtriCure Inc.), targeting autonomic fibres entering posteriorly (*Figure 4*). Finally, transverse RF lesions were applied across the superior and inferior caval veins using the Isolator® Synergy™ Clamp (AtriCure Inc.) to interrupt autonomic fibres traveling along the caval–atrial junctions.

**Figure 4:**
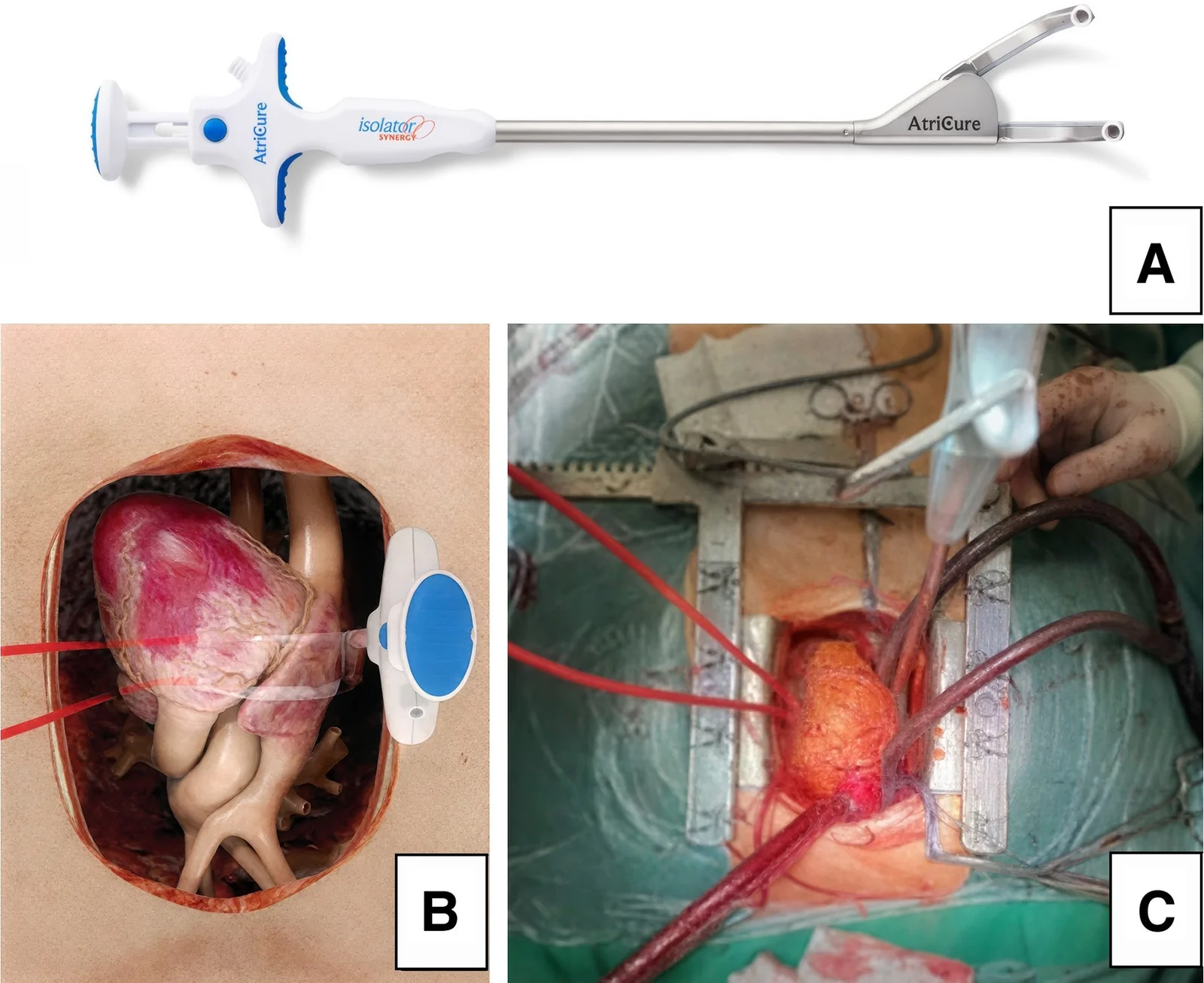
Surgical total cardiac denervation procedure. *(A*) EnCompass® Synergy™ Clamp. *(B*) the use of the EnCompass® Synergy™ Clamp (AtriCure Inc.) as depicted by AtriCure Inc. *(C*) A left atrial box lesion isolating the dorsal left atrium and pulmonary veins was created using the EnCompass® Synergy™ Clamp (AtriCure Inc.), targeting autonomic fibres entering posteriorly. To achieve a complete box lesion, the clamp was applied from both the left and right sides of the heart.

The heart remained *in situ*; no cardiac explantation was performed. The procedure was completed as planned and was well tolerated, with an uncomplicated post-operative course.

### Follow-up and outcomes

Six weeks after surgery the patient reported complete resolution of angina. Post-operative 24-h ambulatory ECG recording showed marked reduction in autonomic modulation (SDNN decreased from 110 to 51 ms). A brief asymptomatic episode of non-sustained ventricular tachycardia occurred, while ST-segment deviations were intermittently present but remained clinically silent, consistent with afferent denervation.

Repeat ^123^I-MIBG scintigraphy demonstrated complete sympathetic denervation (late Heart/Mediastinum ratio 0.82; washout 13%). The patient continues to be free of angina symptoms at the latest follow-up, 9 months after surgery.

## Discussion

This first report of STCD demonstrates the feasibility of a novel surgical strategy for patients with refractory VSA who fail all medical and partial denervation options. Despite prior renal denervation and thoracoscopic sympathectomy, pre-operative HRV and ^123^I-MIBG demonstrated preserved sympathetic innervation, highlighting the limitations of partial autonomic interruption.^5^

STCD targets all major extrinsic cardiac autonomic pathways.^7,8^ The creation of ≥1 cm lesion margins was based on our hypothesis that broader circumferential scarring may reduce sympathetic regrowth; however, this has not been demonstrated in humans and must be confirmed by longer follow-up.^9^ Unlike thoracoscopic sympathectomy, STCD does not involve interruption of the thoracic sympathetic chain. Consequently, complications typically associated with sympathectomy, such as Horner's syndrome, compensatory hyperhidrosis, or anhidrosis, are not expected as direct consequences of STCD.

Post-operative HRV and ^123^I-MIBG findings provided objective confirmation of complete denervation, while the absence of anginal pain despite ischaemic ECG changes suggests effective afferent denervation.^10,11^ Importantly, this dissociation indicates that myocardial ischaemia itself may persist independently of higher neural control, whereas conscious pain perception requires intact afferent pathways. Consequently, patients undergoing STCD may no longer experience typical anginal warning symptoms during myocardial ischaemia. The clinical presentation of ischaemia after STCD remains unknown. This potential risk of silent ischaemia highlights the need for careful patient selection and long-term follow-up. In addition to this concern related to loss of pain perception, severe VSA itself may be associated with life-threatening ventricular arrhythmias, particularly during prolonged or severe episodes of coronary spasm. Therefore, patients presenting with ventricular arrhythmias or cardiac arrest represent a high-risk subgroup requiring careful clinical follow-up and individualized risk assessment.^12^ STCD should therefore currently be considered a last-resort, symptom-directed intervention for highly selected patients with otherwise untreatable symptoms rather than a routine therapeutic strategy.

Further experience is required to evaluate the broader efficacy, durability, safety, and long-term outcomes of this surgical technique.

## Lead author biography


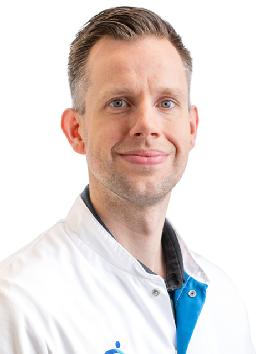
Mitchell Hobers was born and raised in Emmen, the Netherlands. After completing his degree in Physiotherapy, he pursued a pre-master's programme in Medicine. He subsequently worked as a physician in the Department of Cardiology at Treant Scheper Hospital in Emmen and later at the University Medical Center Groningen. In 2020, he began his residency training in Cardiology. During the final year of his training, he prepared the present case report. He currently works as a cardiologist at Nij Smellinghe Hospital in Drachten, the Netherlands.

## Data Availability

All relevant clinical data are included within the manuscript. Additional anonymized data may be made available upon reasonable request.

## Notes

**Consent:** The authors confirm that written consent for submission and publication of this case report including images and associated text had been obtained from the patient, in line with COPE guidance.

## Patient perspective

That after 10 years, I can live without fear and spasms. I can do activities without pain and wake up feeling wonderful without spasms. I no longer struggle with long walks. Finally, a hospital with right doctors and surgeons. The future looks bright.

## Compliance with journal policies

This manuscript meets all ethical, authorship, and submission standards of *European Heart Journal – Case Reports*.
